# Explaining Pre-service Teachers’ Intentions to Use Technology-Enabled Learning: An Extended Model of the Theory of Planned Behavior

**DOI:** 10.3389/fpsyg.2022.900806

**Published:** 2022-07-21

**Authors:** Mingdi Hou, Yigang Lin, Yafei Shen, Hui Zhou

**Affiliations:** ^1^Key Laboratory of Intelligent Education Technology and Application of Zhejiang Province, Zhejiang Normal University, Jinhua, China; ^2^College of Teacher Education, Zhejiang Normal University, Jinhua, China; ^3^Jinhua Institute for Advanced Studies, Jinhua, China

**Keywords:** pre-service teachers, technology-enabled learning, theory of planned behavior, constructivist pedagogical beliefs, ICT competencies

## Abstract

This study proposed an extended theory of planned behavior (TPB) to examine the factors that influence pre-service teachers’ intention to use technology-enabled learning, using constructivist pedagogical beliefs (CPB) and information and communication technologies (ICT) competencies as antecedent variables for attitudes, subjective norms (SNs), and perceived behavioral control. An online study was conducted with a random sample of pre-service teachers from 7 universities in China, and 811 validated questionnaires were obtained. The results showed that the extended TPB model explained 75% of the variance in intention; attitude, SNs, and perceived behavioral control had a positive and significant effect on intention. Furthermore, SNs had a positive and significant effect on attitude and perceived behavioral control. CPB were the antecedent variables for attitude, SNs, perceived behavioral control, and ICT competencies. ICT competencies were the antecedent variable for SNs and perceived behavioral control. Additionally, through multi-group analysis, this study found significant differences in path relationships between the lower and higher-grade groups. The effect of perceived behavioral control on intention diminished with increased grade level. The effect of SNs on perceived behavioral control reduced. The effect of CPB on attitude and perceived behavioral control on intention diminished. The effect of SNs on attitude increased. This study verified that adding the relationship between antecedent variables of theoretical factors and theoretical factors is an effective way to expand TPB and provided a reference for future studies to focus on the related intention of pre-service teachers. Furthermore, it recommends that Chinese universities should eliminate the hindering influence of CPB, ICT competencies, attitudes, SNs, and perceived behavioral control in the process of preparing pre-service teachers. They should also pay attention to the individual differences of students in different grades and the problems that arise in the existing training.

## Introduction

Technology-enabled learning (TEL) is a student-centered approach to technology integration, which regards technology as a cognitive tool that encourages students to construct knowledge actively, solve problems, and develop higher-order thinking (Jonassen, 1995; Ertmer and Ottenbreit-Leftwich, 2013; Watson and Rockinson-Szapkiw, 2021). Moreover, it is an inevitable direction of pre-service teachers’ professional development using deep integration of information technology and education (Tondeur et al., 2017). To train the main force of the future teaching practice, teachers’ educational institutions have invested considerable time and energy to improve pre-service teachers’ information and communication technologies (ICT) competencies (Admiraal et al., 2017; Tondeur et al., 2018). In a study of 16,439 pre-service teachers in 20 teacher education institutions, Wang et al. (2021) found that although post-graduation pre-service teachers improved their ICT competencies, they still entered the real classroom with teacher-centered technology integration, using technology as a tool for information or knowledge transfer rather than as a cognitive partner for students (Huang and Teo, 2021). Therefore, the academic focus was on enabling pre-service teachers to use TEL in their future classrooms (Ertmer and Ottenbreit-Leftwich, 2013). However, pre-service teachers lack the opportunity to implement technology integration in real schools as they are students and cannot teach classes (Teo, 2012). Therefore, researchers focused on how teacher education institutions could increase preservice teachers’ intention to use TEL.

Scholars found that the barriers for teacher technology integration, could be divided into three categories: first-order barriers (e.g., resources, training, and support), second-order barriers (e.g., attitudes and beliefs, and knowledge and skills), and third-order barriers (e.g., design thinking skills and disposition) (Ertmer, 1999; Tsai and Chai, 2012). Hew and Brush (2007) argued that removing first-order barriers would facilitate teachers’ technology integration. However, Watson and Rockinson-Szapkiw (2021) investigated schools that removed first-order barriers (i.e., offered relevant curriculum programs for their pre-service teachers), and found that the relevant courses offered by the schools did not have a significant effect on pre-service teachers’ intention to use TEL. Research subsequently argued that teachers’ beliefs about learning were critical to technology integration (Ajayi, 2009), endorsing Ertmer et al.’s (2012) view that the second-order factors were the gatekeepers of teachers’ technology integration. Moreover, Farjon et al. (2019) found that teachers’ beliefs had a significant effect on intention. Gurer and Akkaya (2021) revealed through a study of 714 pre-service teachers that constructivist pedagogical beliefs (CPB) had a greater effect on intention compared with transmissive pedagogical beliefs. However, Kramarski and Michalsky (2015) found that although pre-service teachers preferred CPB, they had concerns about technology integration (Batane and Ngwako, 2016). Tsai and Chai (2012) argued that if second-order barriers were removed, teachers would continue to use technology in a teacher-centered approach (Huang and Teo, 2021), and only by lowering third-order barriers, would it facilitate integration of technology in a student-centered approach (Tsai and Tsai, 2019). Makki et al. (2018) also suggested that addressing third-order barriers assists teachers to overcome second-order barriers. Therefore, researchers explored third-order barriers by focusing more on Technological Pedagogical Content Knowledge. However, several countries, such as China, have not popularized a systematic curriculum for it, because they emphasized pre-service teachers’ ICT competencies (including pedagogical design competencies) (Xiong and Lim, 2015).

In summary, extant research has not accurately determined a systematic model to elucidate which factors should be eliminated and reduced to preservice teachers’ intention to use TEL. On the one hand, few studies examined CPB and ICT competencies as required factors of TEL (Ertmer and Ottenbreit-Leftwich, 2013), while exploring their effects on the intention to use TEL. On the other hand, few in-depth discussions were conducted, although many scholars found that students’ grade level had an effect on intention (Verdonck et al., 2019; Watson and Rockinson-Szapkiw, 2021).

Therefore, this study selected the classic theoretical model for studying pre-service teachers’ behavioral intentions (BIs), the theory of planned behavior (TPB), as the theoretical foundation. CPB about teaching and learning, and ICT competencies as expansion variables were adopted to construct an expanded model with a high degree of explanation to discover the elements that influence preservice teachers’ intentions to use TEL. A multigroup analysis of structural equation modeling (SEM) was conducted to compare the differences in path relationships among pre-service teachers by grade level. The findings of this study may be applied to suggest rationalized reforms for teacher education in Chinese colleges and universities and bridge gaps in the international research field regarding Chinese research. Moreover, these findings offer innovative ideas for the expansion of TPB.

## Literature Review

### Theory of Planned Behavior and Its Extensions

Theory of planned behavior is derived from the theory of rational behavior (TRA), which is commonly used by scholars to predict people’s behavior and as a reference for people’s behavioral intervention (Ajzen, 1985). TRA is a general model adopted to explain human behavior, which can predict individual decision-making rational conditions (Fishbein and Ajzen, 1975). According to TRA, when people think rationally, BI can best determine human behavior, whereas the factor that can determine BI is behavioral attitude and subjective norm (SN) (Ajzen, 1991). However, when people make decisions, they do not always think rationally, as they also have emotional moments. Therefore, Ajzen (1985) added a third variable that can affect intention, that is, perceptual behavioral control (PBC), thus developing TRA into a new theoretical model TPB.

In TPB, BI is influenced by three core components: attitude, SN, and PBC (Ajzen, 1991), which can explain people’s behavior to a good degree. Armitage and Conner (2001) found through meta-analysis that three core components could explain 39% of people’s BI variance and they had better predictability for BI. However, for a deeper understanding of BI and to design more effective interventions for BI, scholars have typically enhanced the degree of interpretation of intentions by TPB by including extension variables (Taylor and Todd, 1995; Ajzen, 2005). Regarding teacher technology integration, we found that TPB can be expanded using two approaches: (a) by adding expansion variables that influence intention (Sun et al., 2019); and (b) by adding expansion variables that influence core constructs (Cheon et al., 2012; Teo et al., 2016; van Twillert et al., 2020; Duan, 2022). Watson and Rockinson-Szapkiw (2021) utilized the first way to increase the variance totally (*R*^2^ = 63.2%), while including program variables that only explained 8.4% of the variance in intention. Teo et al. (2016) extended the TPB model using the second approach, which improved the predictability of teachers’ intention to use technology (*R*^2^ = 71.7%) and enabled the researchers to determine the key variables that affect the core factors. Therefore, to improve the interpretation of TPB on pre-service teachers’ intention to use TEL, this study selected the second approach to expand TPB.

In addition, researchers have found that correlating core constructs also improve the explanation of intention by TPB (Kim et al., 2013; Pan and Wang, 2018). According to Kim et al. (2013), SN significantly impacted attitude, and people’s perceived SN likely helped them gain a positive attitude toward the BI. Pan and Wang (2018) discovered that SN enhances the establishment of attitudes and PBC, using the “value-belief-norm” theory, highlighting the importance of SN in intention formation. Therefore, this study can explore the relationship between SN and attitudes and PBC in pre-service teachers’ intentions to use TEL and verify whether it enhances the explanatory power of TPB on intentions.

### Expanded Variables of the Theory of Planned Behavior

#### Constructivist Pedagogical Beliefs

Constructivist pedagogical beliefs is a type of teaching belief that can impact pre-service teachers’ intention to use TEL (Ertmer and Ottenbreit-Leftwich, 2013). Teaching beliefs are critical indicators of teachers’ implementation of instruction (Pajares, 1992; Ng et al., 2010) and can be broadly categorized as transmissive or constructivist (Samuelowicz and Bain, 2001). Transmissive teaching beliefs are teacher-centered beliefs about teaching and learning, where the teacher is the authority and supervisor. CPB is a student-centered pedagogical belief that students’ cognitive development needs to be autonomously constructed rather than delivered by the teacher (Chan and Elliott, 2004). Research shows that instructors with CPB are more concerned with their student’s needs and interests while serving as “sage on the stage” rather than “guide on the side” (Kerlinger and Kaya, 1959; Watson and Rockinson-Szapkiw, 2021). Furthermore, they also use technology more frequently than teachers who believe they are teacher-centered, and they also prefer to use technology in a more student-centered way (Tondeur et al., 2017). Teachers with CPB, for instance, create problem-oriented learning environments where students are guided to use existing technology tools to solve problems, thereby developing higher-order thinking and problem-solving skills (Islamoglu et al., 2021). Positive student feedback will further enhance teachers’ intention to use TEL (Ertmer and Ottenbreit-Leftwich, 2013).

#### Information and Communication Technologies Competencies

Information and communication technologies competencies are regarded as preconditions for teacher technology integration, and include the knowledge and abilities that instructors must possess to integrate technology (Bingimlas, 2009). ICT competencies refer to a teacher’s ability to apply a certain technology, such as the teacher’s mastery of computers, educational software, the Internet, and other technologies closely related to teaching (Suarez-Rodriguez et al., 2018). However, Cuhadar (2018) argues that the ability of teachers to use technology alone is inadequate to support the complex teaching behavior of technology integration. They should have the ability to integrate technology and teaching, such as, organizing the classroom using technology, creating a teaching atmosphere, and communicating with parents (Aslan and Zhu, 2017; Bahcivan et al., 2019). Therefore, currently, ICT competencies blend technological and pedagogical competencies (Wang et al., 2021), a third-order barrier that includes “design thinking” (Makki et al., 2018). Teachers with such ICT competencies can dynamically create knowledge and training in the face of ICT advances and their associated pedagogical approaches (Aslan and Zhu, 2017). In other words, teachers with these competencies can master technological resources, design teaching activities, and enhance student experiences to facilitate student learning and develop higher-order thinking in their students (Suarez-Rodriguez et al., 2018).

### Aim and Hypotheses of This Study

This study aims to improve the explanation of TPB on pre-service teachers’ intention to use TEL by adding expanded factors and path relationships between core variables. This study was based on the expansion model which was used to explore the mechanisms of influence between CPB and ICT competencies and intention. It provides rational recommendations for teacher education institutions to eliminate and reduce barriers to technology integration.

The research questions are as follows: (a) what is the distinctive effect of core factors on one another? (b) Through which core components do CPB and ICT competencies have an indirect impact on intent? (c) What are the differences in the paths between the grade level groups? Thus, we developed the hypotheses, reflected below. The prediction of the expanded TPB on intention was tested using SEM.

#### Behavioral Intention

Behavioral intention refers to an individual’s intention to perform a particular behavior. In this study, BI refers to the intention of pre-service teachers to use TEL in class. According to TPB, the more pre-service teachers recognize TEL, the more expectations and pressures they feel regarding their use of TEL. Moreover, the easier they believe TEL is, the more likely they will be willing to use TEL. Therefore, the following hypotheses were proposed:

**H1:** Attitude toward behavior (ATB) has a significant and positive influence on BI.

**H2:** SN has a significant and positive influence on BI.

**H3:** PBC has a significant and positive influence on BI.

#### Attitude Toward Behavior

Attitude toward behavior refers to an individual’s disposition toward performing particular behavior (Davis, 1989) and is the best predictor of pre-service teachers’ intention in TPB (Teo et al., 2016), influenced by outcome beliefs (i.e., individuals feel the outcome of a particular behavior) and value assessments (i.e., evaluating the development of a specific behavior) (Ajzen and Fishbein, 2002). In this study, ATB refers to the extent to which pre-service teachers approve TEL use based on their beliefs and experiences. Han et al. (2017) discovered pre-service teachers by holding more transmissive pedagogical beliefs would have a low intention to use TEL, but CPB would not (Taimalu and Luik, 2019). Liu et al. (2017) included CPB had an indirect effect on intention via behavior and attitude. This may be attributed to the fact that human beliefs are hierarchical, similar to atoms (Rokeach, 1989). Although central beliefs influence peripheral beliefs, attitudes are more marginalized than CPB (Bahcivan et al., 2018) and are influenced by CPB. Based on the above, pre-service teachers who have greater CPB are more likely to have a beneficial effect on TEL. Therefore, the following hypothesis was proposed:

**H4:** CPB has a significant and positive influence on ATB.

#### Subjective Norm

Subjective norm represents the external pressures and expectations that one perceives to perform a particular behavior, mainly influenced by normative beliefs (i.e., the individual’s expectations that others should perform a particular behavior) and compliance motivation (i.e., the individual’s intention to conform to others’ reported expectations) (Ajzen, 1991). Teachers develop stronger intentions when those around them have higher demands and expectations (Huang et al., 2020). Teachers are more likely to perceive their needs from school administration, colleagues, and parents (Sugar et al., 2004). However, pre-service teachers also have a special status among students; this enables them to perceive the views of their teachers and classmates in the current school environment and the views of their colleagues, parents, and students in the future school (Watson and Rockinson-Szapkiw, 2021). Therefore, this study suggests that pre-service teachers’ perceived pressures and expectations from current and future school environments will affect their intention to use TEL.

Subjective norm may significantly affect ATB and PBC (Lu et al., 2019; Xie et al., 2019). In line with this, Xie et al. (2019) concluded that SN is the antecedent of ATB and CPB, and SN significantly affects the formation of ATB and CPB. Lu et al. (2019) argued that Chinese people usually consider face and group consistency, and verified that SN significantly affects the formation of ATB and CPB. Therefore, the following hypotheses were proposed:

**H5:** SN has a significant and positive influence on ATB.

**H6:** SN has a significant and positive influence on PBC.

In addition, SN formation may be influenced by CPB and ICT competencies. Liem and Bernardo (2010) found that epistemological beliefs significantly impact behavior-related beliefs (i.e., attitudes, personal norms, and PBC) in TPB. CPB operates as a derivative belief of epistemological beliefs (Bahcivan et al., 2018). Deng et al. (2014) found that epistemological beliefs significantly impact their beliefs about using ICT through teachers’ pedagogical beliefs. Therefore, this study concluded that CPB would significantly impact behavior-related beliefs about the use of TEL in TPB. In addition, Gieure et al. (2020) found that entrepreneurship ability had a significant impact on college students’ perceptions of whether the people around them support their entrepreneurship. Therefore, the following hypotheses were proposed:

**H7:** CPB has a significant and positive influence on SN.

**H8:** ICT competencies have a significant and positive influence on SN.

#### Perceived Behavioral Control

Perceptual behavioral control is defined as the ease with which a person performs a particular behavior given the resources and opportunities available, as determined by control beliefs (i.e., the individual’s estimate of the importance of the perceived facilitators) and perceived facilitators (i.e., people’s perceptions of the capabilities, resources, and opportunities available) (Ajzen, 1991). The PBC in this study represents the degree of pre-service teachers’ control over the use of TEL, including control over their abilities and control over the surrounding environment and resources.

Constructivist pedagogical beliefs and ICT competencies may influence PBC, which is consistent with the concept of self-efficacy (i.e., a person’s confidence in performing the behavior and the degree of control over the environment) developed by Bandura (1977). Therefore, according to the Self-efficacy Theory, the more competent the preservice teacher is, the greater their self-efficacy. In addition, according to the above, PBC is among the behavior-related beliefs that influence preservice teachers’ use of TEL. Therefore, the following hypotheses were proposed:

**H9:** CPB has a significant and positive influence on PBC.

**H10:** ICT competencies have a significant and positive influence on PBC.

In addition, Bahcivan et al. (2019) found that teachers’ teaching beliefs had a significant impact on ICT competencies, suggesting that pre-service teachers with PBC would consider the differences between students and use technology to meet students’ needs. Therefore, this study hypothesizes:

**H11:** CPB has a significant and positive influence on ICT competencies.

**FIGURE 1:**
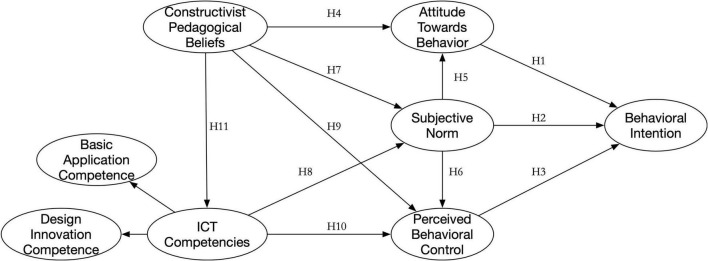
Research model of an extended theory of planned behavior.

These hypotheses resulted in the structural model represented in Figure 1.

## Materials and Methods

### Participants

Participants were from seven universities located in seven different cities in China, which considerably emphasize the development of pre-service teachers’ CPB and ICT competencies by offering a system of related supporting courses. This included three normal universities (with a focus on pre-service teacher training) and four comprehensive universities (with specialized colleges for pre-service teacher training). We used random sampling and collected 1,120 questionnaires. However, 309 had missing answers and were excluded; therefore, 811 valid questionnaires were obtained. The sample included 118 (14.5%) men and 693 (85.5%) women; the mean age of all participants was 20.41 years (SD = 0.56). Of these, 313 (38.6%) were freshmen, 210 (25.9%) sophomores, 220 (27.1%) juniors, and 68 (8.4%) seniors. Participants’ subjects were widely distributed among Math, Language, English, Physics, Biology, Geography, History, Computer, Political, Arts, Physical, and Music.

### Measures

The study used the validated items to explore the expanded TPB based on previous studies. An online questionnaire with two parts was designed for this study. The first part had four questions that collected demographic information about pre-service teachers, including age, gender, education level, and subject. The second part included 7 constructs with 25 questions. The four scales of BI were adapted from Watson and Rockinson-Szapkiw (2021). The distribution of questions by scales is as follows: BI scale: four, ATB scale: three; SN scale: four; and PBC scale: four. First, the scale was selected because this study was intended to be a more in-depth study of BI, based on Watson and Rockinson-Szapkiw (2021). Thus, the sample included pre-service teachers who had received courses related to technology integration. Second, the scale was pre-researched and had good reliability and validity. The CPB scale was adapted from Liu et al. (2017) and has four questions. This scale was chosen because it measures Chinese teachers’ beliefs about teaching and learning, and is relatively mature. All Chinese universities use the Information Technology Application Competency Standards for Primary and Secondary School Teachers (for Trial Implementation) (Ministry of Education of the People’s Republic of China, 2014) as the basic standard for developing pre-service teachers’ ICT competencies. This study proposes an ICT competency scale with six questions based on this policy. Furthermore, the ICT competencies scale was used, which contains two dimensions: essential application competencies (proficiency in the use of ICT) and design innovation competencies (effective integration of teaching and learning using ICT). All scales were measured on a 7-point Likert scale ranging from 1 = strongly disagree to 7 = strongly agree.

As Table 1 shows, the Cronbach’s alpha coefficients for BI (0.983), ATB (0.95), SN (0.967), PBC (0.967), CPB (0.961), and ICT competencies (0.956) exceeded 0.9, implying good internal consistency for all scales, following George and Mallery (2011). In addition, we translated the English scale of this study into Chinese. Thereafter, one English expert and two educational technology experts were invited to verify it. The scale was finalized after several rounds of revisions. The English version is available (Appendix A).

**TABLE 1:** Results of the confirmatory factor analysis.

Constructs	Items	Standardized estimates	Cronbach’s α coefficient	CR	AVE
Behavior intention	BI1	0.963	0.983	0.9835	0.937
	BI2	0.973			
	BI3	0.964			
	BI4	0.972			
Attitude for behavior	ATB1	0.911	0.95	0.9501	0.864
	ATB2	0.953			
	ATB3	0.924			
Subjective norms	SN1	0.937	0.967	0.9673	0.8809
	SN2	0.926			
	SN3	0.951			
	SN4	0.94			
Perceived behavioral control	PBC1	0.928	0.967	0.967	0.8799
	PBC2	0.94			
	PBC3	0.946			
	PBC4	0.938			
Constructivist pedagogical beliefs	CPB1	0.933	0.961	0.9613	0.8613
	CPB2	0.932			
	CPB3	0.94			
	CPB4	0.907			
Basic application competence	BAC1	0.907	0.956	0.8945	0.8092
	BAC2	0.892			
Design innovation competence	DIC1	0.931		0.9697	0.8889
	DIC2	0.946			
	DIC3	0.955			
	DIC4	0.939			

*BI, behavior intention; ATB, attitude for behavior; SNs, subjective norms; PBC, perceived behavioral control; CPB, constructivist pedagogical beliefs; BAC, basic application competence; DIC, design innovation competence; CR, composite reliability; AVE, average variance extracted.*

### Procedures

Owing to the impact of the COVID-19 pandemic, this study administered an online survey. First, we compiled the translated and revised questionnaires into electronic questionnaires and put them on the SO JUMP platform – an online survey tool widely used in China. Second, we randomly selected 50 pre-service teachers offline to fill in the questionnaire and collected their opinions. Based on the survey results, the online questionnaire was improved and revised. Finally, we invited seven universities to participate in the survey. First, we sent the purpose of this study and data, the anonymity guarantee, and the link to the online questionnaire to the university’s counterpart. Second, the participants who met the requirements were identified with their counterparts. Subsequently, the university’s counterpart sent the relevant materials to the eligible pre-service teachers randomly through the university’s official online channel and informed them that participation in the study was voluntary. After 2 weeks, 811 valid questionnaires were obtained on the platform. This study obtained approval from the institutional review board of the research institution and ethical standards in treating human participants were upheld while conducting this study.

This study administered an online survey due to the impact of the COVID-19 pandemic. First, we compiled the translated and revised questionnaires into an electronic version and entered it on the SO JUMP platform-an online survey tool widely used in China. Second, we randomly selected 50 pre-service teachers offline to complete the questionnaire and collected their responses. The online questionnaire was improved and revised, based on the survey results. We invited seven universities to participate in the survey. First, we sent the purpose of this study and data, the anonymity guarantee, and the link to the online questionnaire to the university’s counterpart. Second, we work with our university counterparts to identify research subjects at their schools who meet the requirements. Subsequently, the university’s counterpart sent the relevant materials to the eligible pre-service teachers randomly through the university’s official online channel and informed them that participation in the study was voluntary. We obtained 811 valid questionnaires on the platform after 2 weeks. The institutional review board of the research institution approved the study. Ethical standards in treating human participants were upheld while conducting this study.

## Results

### Descriptive Statistics

Descriptive statistical analysis was performed in this study using SPSS 21.0. We found that the mean values of all 25 items exceeded 4.00 and ranged from 4.86 to 5.98, whereas the standard deviations ranged from 0.932 to 1.287. The skewness and kurtosis indices ranged from −0.961 to 0.198 and −0.337 to 1.890, and did not exceed the | 3| and | 10|, as proposed by Kline (2016), indicating that the study data were standard, conformed to a normal distribution, and suitable for further analysis.

### Model Test

Watson and Rockinson-Szapkiw (2021) used hierarchical multiple regression to explore the association between variables, whereas SEM has the advantage of presenting the path relationships between variables. Therefore, this study used SEM with the maximum likelihood to test the hypothesized model by AOMS 24.0. Two main steps are used to analyze the proposed model: the measurement and the structural model (Anderson and Gerbing, 1988). Additionally, this study compared the differences in path coefficients between grade groups through a multi-group analysis of structural equations.

#### Test of the Measurement Model

First, this study examined the composite reliability (CR), average variance extracted (AVE), and discriminant validity. As shown in Table 1, the CR and AVE were more significant than 0.85, which corresponds with Fornell and Larcker (1981) who proposed that these should be above 0.7 and 0.5, respectively. The factor loadings of all items exceeded 0.8, which met the criterion of >0.6 proposed by Hair et al. (2010). In addition, good discriminant validity requires that the square root of AVE > the correlation coefficients of other constructs, as shown in Table 2. Furthermore, the data showed no multicollinearity between the constructs.

**TABLE 2:** Inter-construct correlations.

	BI	ATB	SN	PBC	CPB	BAC	DIC
BI	**0.968**						
ATB	0.835**	**0.930**					
SN	0.806**	0.876**	**0.939**				
PBC	0.728**	0.764**	0.822**	**0.938**			
CPB	0.688**	0.738**	0.749**	0.707**	**0.928**		
BAC	0.361**	0.377**	0.380**	0.471**	0.391**	**0.900**	
DIC	0.319**	0.317**	0.321**	0.446**	0.291**	0.764**	**0.943**

Diagonal elements in bold are the square root of the AVE. BI, behavior intention; ATB, attitude for behavior; SNs, subjective norms; PBC, perceived behavioral control; CPB, constructivist pedagogical beliefs; BAC, basic application competence; DIC, design innovation competence. **p < 0.01.

Second, several metrics were used to verify the fit of the measurement model: the ratio of the minimum fit function χ^2^ to its degrees of freedom (χ^2^/*df*), whose value of 5.0 or lower is ideal (Wheaton et al., 1977); the Tucker–Lewis index (TLI); the comparative fit index (CFI); the feet index (GFI), whose value greater than 0.90 indicate acceptably (Hair et al., 2010); root mean squared error of approximation (RMSEA), with values less than 0.05 as acceptable, and standardized root mean residual (SRMR), with values less than 0.08 as acceptable (Hair et al., 2010). The CFA confirmed that the measurement model in this study had a good fit [χ^2^/*df* = 2.947, TLI = 0.981, CFI = 0.984, GFI = 0.932, RMSEA = 0.049 (0.045, 0.053)], and SRMR = 0.0182.

#### Test of the Structural Model

The structural model describes the hypothesized relationship between exogenous and endogenous variables, and it achieved a good model fit [χ^2^/*df* = 2.941, CFI = 0.984, TLI = 0.981, GFI = 0.930, RMSEA = 0.049 (0.045, 0.053), SRMR = 0.0225]. Table 3 shows that these results supported all the proposed hypotheses. BI accounted for 75% of the variance explained by ATB, SN, and PBC, with ATB as the strongest predictor of BI (ß = 0.621, *p* < 0.001) and SN as the weakest predictor (ß = 0.105, *p* < 0.01). ATB accounted for 85% of CPB and SN. SN was the most predictive of ATB (ß = 0.792, *p* < 0.001) and accounted for 62% of CPB and ICT competences. CPB was the most predictive of SN (ß = 0.731, *p* < 0.001). PBC accounted for 77% of SN, CPB and ICT competencies, SN was the most predictive of PBC (ß = 0.655, *p* < 0.001) and CPB the weakest predictor (ß = 0.135, *p* < 0.001). In addition, ICT competencies accounted for 17% of CPB (ß = 0.407, *p* < 0.001).

**TABLE 3:** Results of the structural model.

Hypotheses	Paths	Standardized coefficient	*t*	Results
H1	ATB→BI	0.621***	10.542	Supported
H2	SN→BI	0.168*	2.537	Supported
H3	PBC→BI	0.105**	2.714	Supported
H4	CPB→ATB	0.162***	5.741	Supported
H5	SN→ATB	0.792***	25.403	Supported
H6	SN→PBC	0.655***	19.054	Supported
H7	CPB→SN	0.731***	24.706	Supported
H8	ICTC→SN	0.116***	4.128	Supported
H9	CPB→PBC	0.135***	4.079	Supported
H10	ICTC→PBC	0.212***	8.283	Supported
H11	CPB→ICTC	0.407***	10.575	Supported

BI, behavior intention; ATB, attitude for behavior; SNs, subjective norms; PBC, perceived behavioral control; CPB, constructivist pedagogical beliefs; ICTC, ICT competencies. *p < 0.05; **p < 0.01; ***p < 0.001.

### Multigroup Analysis of Structural Equation Modeling

#### Invariance Test of the Measurement Model

We grouped participants by grade level. Freshman and sophomore years were divided into the low-grade group and juniors and seniors into the high-grade group. We set a parameter-free constrained model, Model 1 [χ^2^ = 1330.138, *df* = 518, CFI = 0.974, TLI = 0.970, RMSEA = 0.044 (0.041, 0.047), AIC = 1594.138, ECVI = 1.971] for which the model fit coefficient was good. Thereafter, we set Model 2 with equal factor loadings [χ^2^ = 1346.882, *df* = 536, CFI = 0.974, TLI = 0.971, RMSEA = 0.043 (0.040, 0.046), AIC = 1574.882, ECVI = 1.947], and the model fit coefficient was better. A comparison of Model 1 with Model 2 revealed no statistically significant increase in Chi-square values (Δχ^2^ = 16.744, *p* = 0.541). This indicated that the models were not significantly different, and the measurement model was invariant for different grade groups.

#### Multigroup Comparison

**FIGURE 2:**
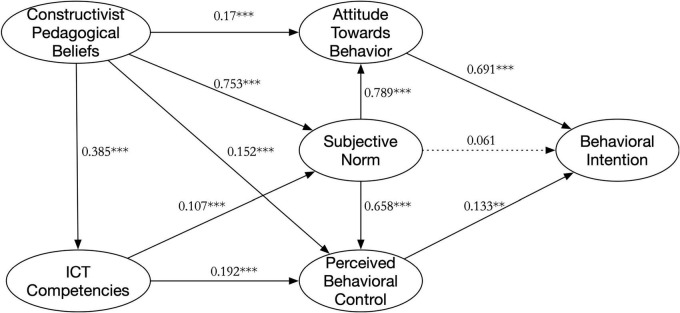
The model of the lower grade group.

**FIGURE 3:**
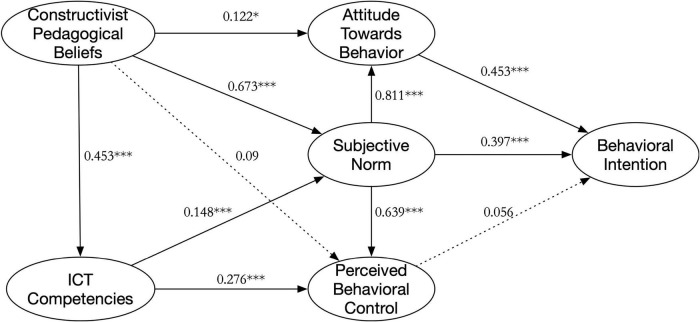
The model of the higher grade group.

Based on Model 2, the path coefficients were set equal to form a measurement path coefficient model, Model 3 [χ^2^ = 1375.010, *df* = 548, CFI = 0.974, TLI = 0.971, RMSEA = 0.043 (0.040, 0.046), AIC = 1579.010, ECVI = 1.952], with a good model fit. Model 2 and Model 3 were compared and the increase in the Chi-squared value (Δχ^2^ = 28.128, *p* = 0.005) was statistically significant, indicating a significant difference between the lower and higher-grade groups in the pathway relationship, Figures 2, 3 show the specific paths for the two grade groups. The critical ratios for differences (CRDs) were used to compare the path coefficients in the model. If the CRD between parameters was greater than 1.96, a significant difference between the two parameters was indicated (Arbuckle, 2004). Table 4 shows there were significant differences between the two groups in the pathway relationships for H3, H4, H5, H6, and H9. Results showed that with increasing grade level, the effect of PBC on BI gradually decreased (H3), CPB on ATB gradually decreased (H4), and CPB on PBC gradually decreased (H9); the effect of SN on PBC gradually decreased (H6), and the effect of SN on ATB gradually increased (H5). In addition, there was no significant effect of SN on BI in the lower grade group (H2); PBC on BI, CPB on PBC in the higher-grade group (H3 and H9).

**TABLE 4:** Standardized path coefficients in SEM or grade level.

Hypotheses	Paths	Grade level		
		Lower level	Higher level	CRD
H1	ATB→BI	0.691***	0.453***	1.925
H2	SN→BI	0.061	0.397***	–0.76
H3	PBC→BI	0.133**	0.056	–2.003
H4	CPB→ATB	0.17***	0.122*	2.586
H5	SN→ATB	0.789***	0.811***	–2.706
H6	SN→PBC	0.658***	0.639***	2.979
H7	CPB→SN	0.753***	0.673***	–0.296
H8	ICTC→SN	0.107***	0.148**	–0.003
H9	CPB→PBC	0.152***	0.09	–2.225
H10	ICTC→PBC	0.192***	0.276***	–1.366
H11	CPB→ICTC	0.385***	0.453***	1.238

BI, behavior intention; ATB, attitude for behavior; SNs, subjective norms; PBC, perceived behavioral control; CPB, constructivist pedagogical beliefs; ICTC, ICT competencies.*p < 0.05; **p < 0.01; ***p < 0.001.

## Discussion

This study examined the factors influencing Chinese preservice teachers’ intention to use TEL by expanding the TPB. The results supported the 11-path hypothesis (H1–H11) in the extended model, which explained 75% of the variance in intention. This was significantly higher than the 39% defined by the original TPB (Armitage and Conner, 2001). Furthermore, through multi-group analysis, we found that there were differences in pathways between the lower and higher-grade groups, with significant differences in five pathways (H3, H4, H5, H6, and H9). These findings are discussed below.

The antecedent variables of BI differed across grade groups. First, ATB had a positive and significant effect in the lower and higher-grade groups and the most significant impact on BI. This finding is consistent with previous findings that pre-service teachers could only prompt a shift in their willingness to use TEL by shifting their attitudes toward it (Ajzen and Fishbein, 2002). Only by deepening their understanding and acceptance of TEL will pre-service teachers use it in the future (Ertmer et al., 2012). Second, there was uncertainty in the effect of SN on BI across grade groups. This finding concurs with Teo et al. (2016). They argued that it had a significant effect on intention only when the system was mandatory for teachers or those who lacked teaching experience. In China, the Confucian culture and collectivism of respecting teachers are deeply rooted (Huang and Teo, 2020), making pre-service teachers pay heed to the teachings of teacher educators and value the views and opinions of those around them. Thus, schools advocating and requiring the active use of TEL will help inexperienced pre-service teachers to develop an intention to use TEL (Teo et al., 2019). There is no significant effect of SN on BI in the lower grade group, which on the one hand indicates that colleges and universities do not have sufficient expectations and requirements for students, and pay more attention to their subject knowledge and professional skills mastery. On the other hand, students who recently entered campus are still relatively unfamiliar with individuals around them, and do not have many channels to receive information about their surroundings. Third, the effect of PBC on BI diminished with increasing grade level (H3). The emergence of this phenomenon may be related to the accumulation of experience of pre-service teachers (Farjon et al., 2019). Students in the lower grades are more likely to accumulate pedagogical knowledge and become proficient in the use of information technology. This accumulation of experience makes them increasingly confident in their use of TEL, which in turn promotes intention. As they enter the higher grades, pre-service teachers begin to have access to real classrooms to observe and practice using TEL, and they find it exceptionally difficult to use (Watson and Rockinson-Szapkiw, 2021). Although schools ostensibly advocate student-centered instruction, teacher-centered test-based education remains the dominant teaching practice to meet students’ academic achievement (Zhao et al., 2015). Moreover, they found using TEL to be a tedious teaching activity that consumed a lot of their time and energy. Thus, pre-service teachers in higher grades are reluctant to use TEL although they have increased control (Watson and Rockinson-Szapkiw, 2021).

SN had a positive and significant effect on ATB and PBC, but the pathway relationship differed significantly between the two groups (H5 and H6). Huang and Teo (2020) argued that SN had an impact on BI because pre-service teachers internalized others’ ideas into their own attitudes and degree of control. The findings of this study support their view. The stronger effect of SN on ATB with increasing grade level indicates that students in higher grades are more likely to internalize the collective will. The weaker effect of SN on PBC with increasing grade level shows that students in higher grades do not necessarily have the confidence to take control of TEL after experiencing practical difficulties using it, although the expectations of others around them increase.

Constructivist pedagogical beliefs was the antecedent variable for ATB, SN, PBC, and ICT competencies in the lower grade group, however, it did not significantly affect PBC in the higher-grade group. Moreover, CPB had significant effects on ATB, SN, and PBC, confirming Deng et al.’s (2014) view. With a deeper understanding of constructivist teaching, pre-service teachers will increasingly recognize TEL and more deeply appreciate the expectations and support of those around them (Liu et al., 2017). The study also found that the effect of CPB on SN was much higher than that on ATB, which is inconsistent with the previous view (Tondeur et al., 2017). This may be related to the influence of cultural context. Triandis (1989) argued that in examining behavioral effects, the role of different cultural contexts needs to be considered. Unlike the Western cultural context, China places more emphasis on collectivism. With collective advocacy and demands, pre-service teachers’ deep understanding of the nature of teaching and learning only serves to better understand the demands and wishes of the collective and does not directly translate into their own behavioral beliefs. Therefore, as the sense of collectivism increases with grade level, the influence of CPB on ATB and PBC diminishes to a certain degree (H4 and H9). However, the significant effect of CPB on ICT competencies also concurs with Bahcivan et al. (2019) that when pre-service teachers hold student-centered beliefs, they are concerned with individual differences of students. Therefore, they enhance their ICT applications to meet the student differences.

Information and communication technologies competencies were the antecedent variables of SN and PBC in both the lower and higher-grade groups. This validates the Self-Efficacy Theory and also supports Gieure et al.’s (2020) findings. External expectations and pressure on an individual are felt more as they become more competent. Chinese pre-service teachers will continue to internalize collectivist intentions and increase their self-efficacy as their competency levels increase.

### Implications

This study explored the factors influencing Chinese pre-service teachers’ intention to use TEL through CPB and ICT competencies as the expanded variables of the TPB model. The results demonstrated that the TPB expanded model had a high degree of explanation for the variance of Chinese pre-service teachers’ intention to use TEL. This verifies that adding the antecedent variables of theoretical factors and the relationship between theoretical factors is an effective way of TPB expansion. Moreover, given the commonality of research variables in different contexts, the expanded TPB model will also provide some usefulness for future scholars to study the blended teaching intention and precision teaching intention of preservice teachers.

This study has some practical contributions. On the one hand, it makes Chinese universities aware that if they want to increase pre-service teachers’ intention to use TEL, it is not only necessary to improve pre-service teachers’ CPB and ICT competencies but also to eliminate ATB, SN, and PBC as barriers to BI. On the other hand, colleges and universities should consider the individual differences between the lower and higher-grade groups. For instance, the higher-grade group is less perceptive of individuals’ views and less able to internalize their ideas into their attitudes. This requires colleges to strengthen the channels of interaction and communication between the junior group and those around them and increase practical activities to help them internalize better. Colleges and universities are aware of the problems that arise in the preparation of pre-service teachers. For example, as the grade level increased, students found more difficulties in using TEL, with hindering effects on the formation of ATB, PBC, and BI; students in the higher grades used their learning of constructivist teaching to understand the collective will, proving that they lacked initiative in TEL.

### Limitations and Conclusion

This study has limitations. First, TEL is a teaching philosophy and method advocated by the state, colleges, and universities. When pre-service teachers completed online questionnaires, they possibly overestimated their beliefs, behaviors, and attitudes. More accurate data may be obtained through observations and interviews. Second, this study used cross-sectional data. More accurate and valuable causal relationships may be found in the same group of pre-service teachers and can be tracked and investigated. Third, the added expansion variables are the beliefs and abilities formed by pre-service teachers under the cultivation of colleges and universities. In future, the antecedent variables that affect ATB can be added from their development.

Although there are limitations, this study draws new conclusions based on previous studies. On the one hand, it constructs an expanded model of TPB with a high degree of explanation of intention through CPB and ICT competencies as antecedent variables of ATB, SN, and PBC under the condition that researchers under-researched the intention of pre-service teachers to use TEL. It explores the relationship between the influencing factors, and provides a model for future researchers to investigate technology integration intentions. On the other hand, through the multi-cluster analysis of SEM, it identifies pathway differences and nurturing issues between the lower and higher-grade groups. For example, the reason for the lack of significant effect of SN on BI in the lower-grade group was that colleges and universities did not make it mandatory; the diminishing effect of PBC on BI and SN on PBC with increasing grade level was because higher-grade students gradually discovered practical difficulties in using TEL, but were unable to self-solve them. There was a diminishing effect of CPB on ATB and PBC with increasing grade level because the higher-grade students adopted the constructivist instruction to understand the collective will and it did not directly contribute to their own internalization. It is recommended that Chinese universities pay attention to the development of all elements of pre-service teachers; focus on the individual differences of students in different grades; and identify the problems in the existing approach.

## Data Availability Statement

The datasets presented in this article are not readily available because the original dataset requires permission from Zhejiang Normal University before it can be made available. Requests to access the datasets should be directed to MH, houmindy@163.com.

## Ethics Statement

The studies involving human participants were reviewed and approved by the Ethics Review Committee of Zhejiang Normal University. The patients/participants provided their written informed consent to participate in this study.

## Author Contributions

MH and YL developed the original idea for the study. MH designed the research and wrote the manuscript. YS and HZ revised it critically for important content. All authors contributed to the article and approved the submitted version.

## Conflict of Interest

The authors declare that the research was conducted in the absence of any commercial or financial relationships that could be construed as a potential conflict of interest.

## Publisher’s Note

All claims expressed in this article are solely those of the authors and do not necessarily represent those of their affiliated organizations, or those of the publisher, the editors and the reviewers. Any product that may be evaluated in this article, or claim that may be made by its manufacturer, is not guaranteed or endorsed by the publisher.
